# Biomarcadores em Doenças Cardiovasculares: O Papel da Fetuína-A

**DOI:** 10.36660/abc.20210980

**Published:** 2022-01-01

**Affiliations:** 1 UNESP Faculdade de Medicina de Botucatu Departamento de Clínica Médica Botucatu SP Brasil Departamento de Clínica Médica - Faculdade de Medicina de Botucatu (UNESP), Botucatu, SP - Brasil; 2 Universidade Federal de Mato Grosso do Sul Instituto Integrado de Saúde Campo Grande MS Brasil Instituto Integrado de Saúde, Universidade Federal de Mato Grosso do Sul (UFMS), Campo Grande, MS - Brasil

**Keywords:** Doenças Cardiovasculares/mortalidade, Fetuína-A, Calcificação Vascular, Infarto do Miocárdio, Acidente Vascular Cerebral, Inflamação, Prognóstico

As doenças cardiovasculares (DCV) possuem altas taxas de incapacidade e mortalidade em todo o mundo, representando sério problema de saúde pública.^1^ Atualmente, muitas pesquisas têm se intensificado na procura por biomarcadores associados às DCV.^2,3^

A fetuína-A é uma glicoproteína multifuncional, predominantemente expressa no fígado, que apresenta diferentes mecanismos de ação como, por exemplo, modulação da calcificação vascular, da resposta à inflamação sistêmica, do metabolismo ósseo e da atividade da insulina.^4,5^ Na última década, pesquisadores têm avaliado o papel da fetuína-A no desenvolvimento de DCV e como biomarcador do prognóstico de pacientes com diferentes DCV.^6^ Baixas concentrações plasmáticas de fetuína-A foram associadas a maior gravidade de calcificação de artérias coronárias.^7^ Adicionalmente, a concentração plasmática de fetuína-A foi estatisticamente menor em pacientes com infarto agudo do miocárdio do que em indivíduos saudáveis.^8^ Em contraste, Vörös et al.,^9^ mostraram que indivíduos com infarto do miocárdio prévio apresentaram concentrações plasmáticas significativamente maiores de fetuína-A em relação a indivíduos não infartados. Altas concentrações de fetuína-A também foram associadas a maior risco de acidente vascular isquêmico.^10^ Esses dados mostram que o envolvimento da fetuína-A com DCV ainda não é totalmente compreendido, pois tanto níveis mais baixos quanto mais elevados foram associados com presença ou risco aumentado para DCV. Assim, estudos que abordem o tema e avaliem o papel da fetuína-A como possível biomarcador para DCV são importantes atualmente.

Nesta edição dos Arquivos Brasileiros de Cardiologia, o estudo de Çakır et al.,^11^ revelou dados interessantes sobre a associação entre concentração sérica de fetuína-A na fase aguda do infarto do miocárdio com supradesnivelamento do segmento ST e prognóstico de longo prazo. Os autores acompanharam 180 pacientes por período médio de 10 anos. A amostra do estudo foi dividida em dois subgrupos de acordo com a concentração sérica de fetuína-A (≤ 288 μg/mL e > 288 μg/mL), quantificada durante a internação. O acompanhamento foi realizado por contato telefônico após a alta hospitalar. O estudo mostrou que menor concentração de fetuína-A foi associada a pior prognóstico, com mortalidade global e por DCV significativamente maior no grupo com concentração menor de fetuína-A (44% vs 24%, p=0,005; e 48% vs 31%, p=0,022, respectivamente). Além disso, a concentração de fetuína-A foi inversamente correlacionada à concentração sérica da proteína C reativa ultra-sensível e, curiosamente, apresentou correlação direta com a glicemia de jejum. Como variável categórica e contínua, a concentração de fetuína-A foi um preditor de mortalidade cardiovascular independentemente de fatores de risco tradicionais.

Após o infarto agudo do miocárdio, o processo inflamatório crônico que se estabelece está intimamente relacionado com remodelamento adverso, disfunção ventricular e insuficiência cardíaca.^12^ Uma das hipóteses dos autores é que maior concentração de fetuína-A possa ter papel benéfico após o infarto na regulação da inflamação e, consequentemente, no processo de cicatrização miocárdica. A relação entre fetuína-A e resposta inflamatória já foi descrita em outras condições. Em modelo experimental de sepse, níveis circulantes de fetuína-A foram menores na presença dos mediadores inflamatórios IFN-γ, TNF-α e HMGB1 e a suplementação exógena de fetuína-A diminuiu drasticamente a concentração desses mediadores, o que sugere seu papel protetor na inflamação.^13^ Similarmente, em estudo *in vitro*, macrófagos estimulados com lipopolissacarídeo exibiram alta produção de IL-1β e óxido nítrico, componentes chave durante a inflamação. Esse quadro foi significativamente revertido pela adição de fetuína-A às culturas macrofágicas.^14^ Outros autores também verificaram a participação da fetuína-A na regulação da resposta inflamatória e associação com melhor prognóstico durante isquemia cerebral.^15^

Os achados de Çakir et al.,^11^ devem ser confirmados em estudos futuros com tamanho amostral maior e mensurações da fetuína-A em diferentes momentos ao longo do tempo para que seu valor prognóstico para doenças cardiovasculares possa ser melhor estabelecido.
