# Supplementary material for: Biomarcadores em Doenças Cardiovasculares: O Papel da Fetuína-A
Source: Arq Bras Cardiol. 2022 Jan 1;118(1):22–3. [Article in Portuguese] doi: 10.36660/abc.20210980 (PMC8959047; doi:10.36660/abc.20210980)
Supplement: Translation

# Translation: Biomarkers in Cardiovascular Disease: The Role of Fetuin-A

**Affiliations:** 1 Botucatu SP Brazil Departamento de Clínica Médica - Faculdade de Medicina de Botucatu (UNESP), Botucatu, SP - Brazil; 2 Campo Grande MS Brazil Instituto Integrado de Saúde, Universidade Federal de Mato Grosso do Sul (UFMS), Campo Grande, MS - Brazil

**Keywords:** Cardiovascular Diseases/mortality, Fetuin-A, Vascular Calcification, Myocardial Infarction, Stroke, Inflammation, Prognosis

Cardiovascular disease (CVD) represents a serious health public concern worldwide due to its high rates of morbidity and mortality.^1^ Extensive research has been performed in the search for biomarkers associated with CVD.^2,3^

Fetuin-A is a multifunctional glycoprotein, predominantly expressed in the liver, which modulates the function of several cells and systems, such as vascular calcification, systemic inflammatory response, bone metabolism, and insulin activity.^4,5^ In the last decade, researchers have evaluated the role of fetuin-A in the development and prognosis of patients with different CVDs.^6^ Lower plasma levels of fetuin-A were associated with a higher severity of coronary artery calcification^7^ and its plasma levels were lower in acute myocardial infarction patients than in healthy individuals.^8^ By contrast, Vörös et al.^9^ reported that individuals with a previous infarction have a significantly higher plasma concentration of fetuin-A than healthy controls. High concentrations of fetuin-A were also associated with a higher risk of ischemic stroke.^10^ These data show that the involvement of fetuin-A with CVD is not completely understood, as both lower and higher levels of fetuin-A were associated with a higher risk or presence of CVD. Thus, it is important to carry out studies that address this topic and highlight fetuin-A as a possible biomarker of CVD.

In this issue of the ABC, the study conducted by Çakır et al.^11^ revealed interesting data on the association between fetuin-A serum levels during acute myocardial infarction with ST-segment elevation and long-term prognosis. The authors included 180 patients, which were divided into two subgroups, according to the fetuin-A serum levels (≤ 288 μg/mL and > 288 μg/mL) assessed during hospitalization. The individuals were followed up by telephone contact over a mean period of 10 years. The study showed that the lower concentration of fetuin-A was associated with a worse prognosis with total and CVD mortality rates significantly higher in the group with the lower fetuin-A concentration (44% vs 24%, p=0.005; and 48% vs 31%, p=0.022, respectively). Furthermore, fetuin-A plasma levels were inversely correlated with ultra-sensitive C-reactive protein and, surprisingly, directly correlated with fasting glucose. As a categorical and continuous variable, fetuin-A was a predictor of cardiovascular mortality, regardless of the traditional risk factors.

The chronic inflammatory process that follows the acute myocardial infarction is closely related to adverse remodeling, ventricular dysfunction, and heart failure.^12^ Therefore, the authors hypothesized that the higher fetuin-A concentration is favorable for patients after acute infarction due to its role in modulating inflammation and improving the healing process. The relationship between fetuin-A and inflammatory response has been described in other conditions. In a sepsis model, circulating levels of fetuin-A were reduced in the presence of the inflammatory mediators IFN-γ, TNF-α, and HMGB1. Interestingly, the exogenous supplementation of fetuin-A drastically decreased the concentration of the mediators, suggesting its protective role in inflammation.^13^ Similarly, in an *in vitro* study, lipopolysaccharide-stimulated macrophages exhibited a high production of IL-1β and nitric oxide, key components during inflammation. This condition was significantly reversed by the addition of fetuin-A to the macrophage cultures.^14^ Other authors have also demonstrated the participation of fetuin-A in the regulation of the inflammatory response and its association with a better prognosis during cerebral ischemia.^15^

The findings of the study by Çakir et al.^11^ should be confirmed in future studies with larger sample sizes and measurements of fetuin-A at different time points to better establish the prognostic value of fetuin-A in cardiovascular disease.
